# Valorization and Application of Fruit and Vegetable Wastes and By-Products for Food Packaging Materials

**DOI:** 10.3390/molecules26134031

**Published:** 2021-07-01

**Authors:** Banu Bayram, Gulay Ozkan, Tina Kostka, Esra Capanoglu, Tuba Esatbeyoglu

**Affiliations:** 1 Department of Nutrition and Dietetics, University of Health Sciences, Uskudar, 34668 Istanbul, Turkey; banu.bayram@sbu.edu.tr; 2 Department of Food Engineering, Faculty of Chemical and Metallurgical Engineering, Istanbul Technical University, Maslak, 34469 Istanbul, Turkey; ozkangula@itu.edu.tr (G.O.); capanogl@itu.edu.tr (E.C.); 3 Institute of Food Science and Human Nutrition, Department of Food Development and Food Quality, Gottfried Wilhelm Leibniz University Hannover, Am Kleinen Felde 30, 30167 Hannover, Germany; kostka@lw.uni-hannover.de

**Keywords:** biopolymers, biocomposites, edible films and coatings, active packaging, intelligent packaging, food waste

## Abstract

The important roles of food packaging are food protection and preservation during processing, transportation, and storage. Food can be altered biologically, chemically, and physically if the packaging is unsuitable or mechanically damaged. Furthermore, packaging is an important marketing and communication tool to consumers. Due to the worldwide problem of environmental pollution by microplastics and the large amounts of unused food wastes and by-products from the food industry, it is important to find more environmentally friendly alternatives. Edible and functional food packaging may be a suitable alternative to reduce food waste and avoid the use of non-degradable plastics. In the present review, the production and assessment of edible food packaging from food waste as well as fruit and vegetable by-products and their applications are demonstrated. Innovative food packaging made of biopolymers and biocomposites, as well as active packaging, intelligent packaging, edible films, and coatings are covered.

## 1. Introduction 

Food waste and food by-product generation is a significant problem that causes undesirable environmental, economic, and social effects. Reducing food waste is a common aim all over the world. There is a great awareness of the severity of the problem; thus, the EU and many other countries promote action plans to decrease food wastes, such as the Farm to Fork Strategy, Circular Economy Action Plan, and EU Waste Legislation. Reduction of food waste could be an important solution to decrease production costs and lead to more efficient food systems. Furthermore, more environmentally sustainable food systems can be created, and food security and nutrition can be improved through waste reduction [1]. 

In recent years, waste valorization has gained great interest to pursue a circular economy that refers to waste reduction and efficient waste management. The prevention and reduction of food waste are the main targets of governments and the food sector. Production of value-added products from wastes such as fuels, materials, and chemicals is the main action in waste valorization contributing to the circular economy. Valorization of food waste from industrial food processing provides many economic, social, and environmental benefits, such as the production of value-added products in different application fields such as organic fertilizers, animal feed, biofuels, and electricity [2,3]. In the last decade, many studies have been conducted for the valorization of food wastes to produce food ingredients, functional foods, nutraceuticals, pharmaceuticals, and cosmeceuticals as they contain many health promoting bioactive compounds such as polyphenols, proteins, lipids, vitamins, and dietary fiber that may improve nutritional, functional, and technological properties of the food products [4,5,6,7]. As a next eco-conscious step of waste valorization, recent studies have demonstrated that the advances in food packaging technology may be an efficient solution to reduce the amount of food waste and by-products through the utilization of waste- and by-product-derived natural materials in the food packaging industry [8,9]. By doing so, global pollution by microplastics could be reduced as functional packaging material becomes available. For instance, by integrating antioxidant compounds obtained from food waste into packaging material, food products become safer and more resistant against spoilage processes. Another possibility of functional packaging could be the use of pH or heating-sensitive colorants, enabling the observation of food quality by the consumer. In this review, recent studies of designed packages, their benefits, and the underlying mechanisms, e.g., functional activities, are discussed. 

## 2. Food Packaging Materials

The global demand for packaging was approximately USD 974 billion in 2018. The demand was the highest in Asia with 40% followed by North America with 21% [10]. According to Food and Agriculture Organization (FAO), the food packaging market has an enormous economic value in the packaging industry, with USD 15.4 billion [11]. It is expected that the value of smart packaging will reach USD 26.7 billion by 2024 followed by intelligent and active packaging systems [12]. Basically, shelf-life extension of food products is the first aim of food packaging. Different packaging materials are used to protect the food content, maintain food quality and safety until it is consumed, and prevent undesirable reactions [13]. The packaging material has to allow controlled respiration, maintain polymer structure against mechanical damage, prevent microbiological and chemical spoilage of food, and act as a selective gas and water vapor barrier [14]. The most widely used materials in the food packaging industry are glass, plastics, metals, and paper. As mechanical properties of materials are important for food protection, mostly flexible and rigid synthetic packaging materials are preferred [15]. Synthetic petrochemical plastics are the most popular packaging materials, including polyethylene terephthalate (PET), polyethylene (PE), polypropylene (PP), polyvinyl chloride (PVC), and polystyrene (PS) [13]. Petrochemical plastics are cost-effective materials, while being excellent barriers for oxygen and aroma compounds, having tensile and tear strength. Furthermore, being soft, light, and transparent are the characteristics that contribute to their preference [8,16]. 

Despite their technological benefits, packaging materials are great sources of waste generated in the world. In 2018, approximately 77.7 million tons of packaging waste was generated. The packaging waste generated was estimated to be 174 kg per inhabitant in the EU. A rise of 6.7 million tons was observed in the amount of generated packaging materials in the ten years from 2008 to 2018, corresponding to an increase of 9.4%. In the EU, the highest packaging waste was generated from paper and cardboard by 40.9% (31.8 million tons), followed by plastics and glass by 19.0% (14.8 million tons) and 18.7% (14.5 million tons), respectively [17].

Petrochemical plastics have a poor water vapor transmission rate and detrimental effects on the environment, as they are not recyclable, renewable, biodegradable, or compostable. Actually, the recyclability rate of plastic packaging materials is 14% [8,16]. Due to this incomplete recycling, they pollute the environment and harm human health through accumulation of non-degradable plastics and the formation of secondary microplastics and hazardous chemicals during manufacturing and use [18]. In 2015, packaging accounted for 36% of plastic production, which was estimated at 400 million tons. Approximately 60% of the plastic production was related to the food packaging industry [19]. Regulatory recycling actions have been applied for packaging plastics to reduce the environmental effect of petrochemical plastics [20]. Due to the awareness of consumers on food safety, health, and nutritional value of food products, the substitution of synthetic polymers by biopolymers and the development of sustainable food packaging systems will ensure reduction of environmental impacts and waste generation. Accordingly, in recent years the development of biodegradable polymers obtained from plant materials, which may replace petrochemical plastics, has increased. 

Biodegradable packaging using biopolymer materials offers some advantages, such as being inexpensive, non-toxic, and transparent as compared to plastics. They have film-forming abilities and act as a carrier for antimicrobial and antioxidant compounds [21]. Furthermore, they are resistant to mechanical damage, and they have water vapor permeability that is useful for packaging of fresh fruit and vegetables. Biodegradable packaging has water absorption capacity and low friction coefficient, and it also acts as a barrier to odors, aromas, fats, and oils [22]. In recent years, it has become a promising trend to add natural additives, extracts, and food processing waste products, including phenolic acids, tannins, proanthocyanidins, or flavonoids, in order to improve food packaging performance.

Through the use of recycled materials and renewable resources as raw materials, reuse, recycling, and biodegradation of packaging materials, the sustainability of food packaging can be achieved. Many packaging innovations have been developed using novel bio-based materials with improved performances in order to increase the packaged product quality, extending the shelf-life and decreasing food waste [23]. Among different packaging applications, the integration of antioxidant acting compounds and/or spoilage indicators in packaging systems are reported to have great potential to reduce food wastes [9]. 

Significant attention has been given in recent years to find alternative polymer materials as added-value novel packaging materials through fruit and vegetable waste valorization. Accordingly, the application of fruit and vegetable wastes and by-products as food packaging materials seems to have two-sided effects. This would both reduce the amount of food packaging waste and the amount of fruit and vegetable-based waste and by-products. 

## 3. Fruit and Vegetable Industry Wastes and By-Products

According to the report of FAO [19], 1.3 billion tons of food waste and food loss has been generated annually. This huge amount of waste and loss may occur at different stages of food processing, such as pre-harvest, harvest, on farm post-harvest, transport, storage and distribution, processing and packaging, retail, and public household consumption [24]. Roots, tubers, and oilseeds are the most important plant-based sources for food waste generation with 25%. Fruits and vegetables are the second important source with 21%, followed by cereals and pulses with 14% [19]. The cost of food waste and loss was estimated at USD 936 billion globally [25]. The agri-food industry generates a significant amount of waste and by-products, contributing to 40–50% of the total discard from different parts of plant sources such as peels, pulps, skins, pomaces, shells, roots, stems, stones, leaves, and seeds [26]. 

In recent years, production of fruit and vegetables has been increasing in order to meet consumer demand. Approximately 0.9 billion tons of fruits and more than 1 billion tons of vegetables were produced in 2017 [27]. Citrus, watermelons, banana, grapes, apples, and mangoes are the most produced fruits. In terms of vegetables, the production rate is the highest for potatoes, tomatoes, onions, cucumbers, and cabbages [26]. Among different fruit and vegetable types, the highest amount of waste is produced from mango (60%), followed by citrus fruits (50%), passion fruit (45%), peas (40%), pineapple (33%), and pomegranate (40%). Furthermore, banana, apple, grape, potato, and tomato are other important waste sources [28,29]. 

The peels, pomace, and seeds of fruit and vegetables are important sources of bioactive compounds such as proteins, dietary fibers, colorants, and phytochemicals. These phytochemical compounds show a variety of beneficial health effects, including antioxidant, antimicrobial, anti-inflammatory, anti-diabetic, anti-carcinogenic, and cardioprotective effects [7,28,30,31,32].

The peel of banana, mango, avocado, citrus, and apple and pomace of grape are important sources of dietary fiber that varies between 51 and 70% [33,34,35]. On the other hand, tomato and carrot pomaces contain high amounts of fiber corresponding to 50% and 64%, respectively [28]. 

Peel and seeds of fruit and vegetables contain high levels of phenolic compounds, including phenolic acids, flavonoids, and tannins. Most of the by-products contain higher amounts of phenolics compared to their flesh part. For example, mango peel was found to have higher total phenolic content (92.6 mg gallic acid equivalents (GAE)/g) than its flesh (27.8 mg GAE/g), both at ripe and unripe stages [36]. Phenolic content also depends on the part of wastes. For example, avocado peel contains higher phenolic content than its seed and pulp [37]. Pectin is another value-added compound obtained from wastes and may play a role as polymeric matrix for active packaging [38]. Pectin has gel-forming ability and act as a thickener, stabilizer, and texturizer. It is compatible with proteins, lipids, and polysaccharides, and it improves the antioxidant and antimicrobial activities of incorporated materials into active packaging [39]. On the other hand, starch is also considered as a valuable material for food packaging as a biodegradable polymer with unique characteristics [40].

## 4. Food Packaging Innovation 

Food packaging obtained from fruit and vegetable wastes and by-products is of great interest due to their unique characteristics. When these wastes and by-products are applied to food packaging systems, they provide many advantages such as increased antioxidant activity, antimicrobial activity, improved mechanical properties, and improved quality of protected food products. There are several studies on the incorporation of these wastes in biopolymers, biocomposites, active packaging systems, intelligent packaging systems, and edible films and coatings to test their applicability for improved packaging characteristics. Some examples of applications of fruit and vegetable wastes and by-products in different food packaging systems are given in Table 1.

### 4.1. Biopolymers

Due to reasons including the current global consumption of plastics and the regaining of biopolymers to utilize them in the fabrication of biodegradable materials, there has been increasing interest to utilize alternative raw materials from agricultural and food processing wastes in food packaging applications [56]. Biopolymers, called renewable polymers, can be classified into four categories: (i) biomass-based polymers, particularly from agro-resources, including polysaccharides (starches, lignocellulosic products, pectins, gums), lipids and protein (casein, whey, collagen/gelatin, zein, soy, and gluten), (ii) polymers obtained by microbial conversion (poly(hydroxyl alkanoates) (PHAs)), (iii) polymers chemically synthesized using monomers obtained from agro-resources (poly(lactic acid) (PLA)), and (iv) polymers obtained by chemical synthesis from fossil resources (aliphatic co-polyesters, aromatic co-polyesters) [57]. Among others, in this review we focused on the use of fruit and vegetable by-products including starches, cellulose derivatives, and pectin.

Starch has been considered as one of the most promising polymeric hydrocarbons and is composed of a mixture of two types of glucose polymers: 20–25% amylose, and 75–80% amylopectin. Amylose molecules form a helix structure by the bond angles in the range of 200–20,000 glucose units. Amylopectin is a branched molecule, in which linear chains of α(1→4)-linked glucosyl units are joined to each other by α(1→6) branches, containing up to two million glucose units [58]. The linear amylose exhibits mostly an amorphous structure, whereas the branched amylopectin has crystalline areas. Starch occurs as a stored carbohydrate in plants like rice, corn, cassava, potatoes, and cereal grain, which is in the form of granules, in different sizes and compositions [59]. Starch can be used as a filler, coating, or film due to its easy availability, abundance, biodegradability, lower cost, and good mechanical properties, providing a mixture with conventional polymers and ease of processing by the equipment used in conventional polymer processing, including extrusion and injection molding [60]. In addition to these, starch presents thermoplastic behavior. It can be fabricated in gelatinizing granular forms, with varying viscosities, water solubility, and water absorption by modifying the amylose/amylopectin ratio, moisture/plasticizer content, temperature, and pressure in the extruder [61]. On the other hand, it is not possible to use thermoplastic starch widely because of its moisture sensitivity and poor mechanical properties. They are generally used in polymer blends or nanocomposites, such as Mater-Bi (from Novamont, Novara, Italy), Bioplast^®^ (from Biotec GmbH, Gütersloh, Germany), and NOVON^®^ (from NOVON International, Buffalo, NY, USA) [16,62]. Moreover, starch can also be utilized in soluble compostable foams, expanded trays, or disposable dishes. Biopur^®^ (from Biotec GmbH), Eco-Foam^®^ (from National Starch & Chemical, Bridgewater Township, NJ, USA), and Envirofill^®^ (from Norel, Waltham, MA, USA) have product lines of thermoplastic starch [16].

Cellulose is one of the most plentiful biopolymers present in nature, consisting of repeating units of D-glucose linked via β-1,4 glycosidic bonds [63]. In the cell wall of plants and algae, cellulose occurs as a structural constituent, and wood is the major source of cellulose with a 40–50% ratio by weight. It is biodegradable, recyclable, renewable, biocompatible, and comparatively highly endurable [64]; therefore, it can be used in coatings, laminates, films, additives in construction products, nanocomposites, and pharmaceuticals [65]. The structure of cellulose is related to the isolation process, quantity of inter- and intramolecular hydrogen bonds, and degree of polymerization and crystallinity [66]. Due to its high crystallinity and insolubility in water, it is not a suitable candidate for film formation. Therefore, it is transformed into cellophane film, exhibiting better mechanical properties. Furthermore, cellophane is coated to enhance its humidity barrier structures to be used for bakery products, processed meat, cheese, and candy. On the other hand, due to its non-thermoplastic nature, cellophane is not suitable for heat sealing [67]. Furthermore, there have been several studies about cellulose-based blends such as low-density polyethylene/cellulose or low-density polyethylene/cellulose/chitin, chitosan to improve the biodegradability of cellulose [68].

Pectin, widely present in agricultural waste materials, can be altered by demethylation in the presence of calcium ions to form edible films [69]. Due to the fact that the moisture resistance of pectin films is quite low, it is limited to being utilized in food packaging. Thus, various attempts have been implemented on blending pectin and other biodegradable materials such as chitosan/pectin laminated films [70] and pectin/poly (vinyl alcohol) blends [71].

### 4.2. Biocomposites

Due to concerns about environmental issues, changes in the climate pattern, as well as global warming, the production of biopolymer-based green composites and reinforcement with natural fibers have gained great attention. Thereby, the use of eco-friendly bio-based materials, including the valorization of agricultural wastes, has been currently the subject of various investigations [14,72]. Furthermore, countries such as France, Costa Rica, and Brazil have decided to withdraw the non-biodegradable plastics in the composition of disposable materials until 2020, 2021, and 2028, respectively [73].

Composite materials reinforced with synthetic fibers such as aramid, glass, and carbon fibers have a number of desirable properties including high strength, good wear resistance, reliability, and high fatigue life. However, they have some serious drawbacks like high cost and non-biodegradability [74]. On the other hand, natural or bio-based fibers with biopolymer matrices are characterized by low density and renewability as well as low manufacturing and disposal costs [75]. Natural fibers are classified into three main categories: vegetable, animal, and mineral fibers. Fibers from vegetables or plants are then categorized as leaf fibers such as banana and pineapple; bast-based fibers like kenaf, jute, flax; and fibers from seed, including cotton, rice husk, and coir. Fibers are constituted primarily by holocellulose (cellulose, hemicellulose) and lignin as well as lower amounts of sugars, starch, proteins, and ash [76]. Chen et al. [77] investigated the potential implementation of cellulose nanocrystals derived from potato peel waste as a reinforcement and vapor barrier additive in polyvinyl alcohol and thermoplastic starch composites. Results indicated that there was an enhancement in tensile modulus around 19 and 33% in starch composites as well as 38 and 49% in polyvinyl alcohol composites by the reinforcement with 1 and 2% cellulose nanocrystals, respectively. Additionally, while a significant decrease was obtained in the water vapor transmission measurements for the PVA (polyvinyl alcohol) composite, there was no effect for the thermoplastic starch composite [77]. Alwani et al. [78] explored the kinetics of thermal decomposition of different agricultural waste fibers including banana pseudostem, pineapple leaf, and sugarcane bagasse fibers. According to the thermogravimetric analysis, these fibers showed two or three steps of mass loss due to the decomposition of cellulose, hemicellulose, and lignin. Furthermore, these related fibers were thermally stable up to 200 °C. The activation energies were calculated as 157, 137, and 133 kJ/mol for banana pseudostem, pineapple leaf, and sugarcane bagasse fibers, respectively [78]. The efficient use of waste adds value to agro-industrial waste. In this regard, Vannini et al. [79] aimed to valorize the solid fraction of sweet potato, rich in starch and fibrous components (pectin, cellulose, hemicellulose and lignin), after the industrial extraction of starch. Then, sweet potato residue was intended to be utilized for the preparation of new biocomposites designed for food packaging applications. For this purpose, this fraction was added to poly(3-hydroxybutyrate-*co*-3-hydroxyvalerate) in various amounts ranging from 5 to 40 wt % by melt mixing at two different processing temperatures and times: at 200 °C for 5 min, or at 180 °C for 6 min. It was reported that the composites were in a semicrystalline structure and thermally stable up to 260 °C. Moreover, a good dispersion of the sweet potato residue into the bio-polymeric matrix was obtained, presumably owing to the hydrogen bond interactions between the phases. In addition to these, a lower but not significant interfacial adhesion was detected by scanning electron microscopy. Findings resulted in a valuable bio-alternative to the materials commonly used in the packaging industry [79]. In another study, mango seeds from agro-industry by-products were used to fabricate a green biocomposite for rigid packaging. Various biocomposites were produced by using an extrusion/injection processing technique. Biocomposites were formulated with poly (lactic acid) matrix and mango seed by-products, including integument and kernel varying them by 10 and 20 wt %. According to the results of characterization analysis, kernel in the biocomposite of poly (lactic acid) + 20 wt % kernel presented thermal degradation, whereas other compositions kept their morphology. Moreover, the most important progress in mechanical and barrier properties of biocomposites was obtained with the formulation containing 20 wt % integument, suggesting poly (lactic acid)/integument or kernel blends as a new source for biomaterial-based food packaging systems [80].

### 4.3. Edible Films and Coatings

In addition to the increasing demand for recycling and waste reduction, foods should be of high quality and safe by possessing a long shelf-life [51,81,82]. Such quality-improving effects may also be achieved by edible food packaging. For instance, edible films and coatings could decrease the loss of aroma or otherwise prevent the migration of disturbing flavors of the outer atmosphere into the food [81,83]. Thus, by using an alginate film-forming solution, based on hydrophilic carbohydrates isolated from brown seaweed, the oxygen permeability could be reduced, resulting in a longer lasting aroma and water preservation [83]. Moreover, the entire shelf-life of food products can be extended by reducing the oxygen permeability, which otherwise may induce spoilage reactions or discoloration by enzymatic browning mechanisms [81,84]. Similar to the oxygen exclusion, the uptake of fat can be minimized by edible films and coatings, supporting the actual trend of healthier consumption. Dragich et al. [85] analyzed the efficiency of a reduced fat uptake by coating chicken breast filets with different mixtures of wheat flour and whey protein isolates before frying. On one hand, the whey protein coating led to a significantly lower fat content, which was reduced by over 30% compared to the chicken filets without such a coating. On the other hand, the whey protein isolate had no noticeable effect on the color and texture of the food [85]. Another application of edible films is to improve the appearance or visual quality of food with high gloss coatings, especially on fruits and vegetables [81,86]. While whey protein isolates, shellac, dextrin, and zein are suitable materials for a gloss coating, zein and dextrin are more sensitive to humidity and becoming de-adhered and cracked. In contrast to this, the glossy effect of a whey protein coating could be further enhanced by adding lipids in the form of milk fat [86].

**Figure 1:**
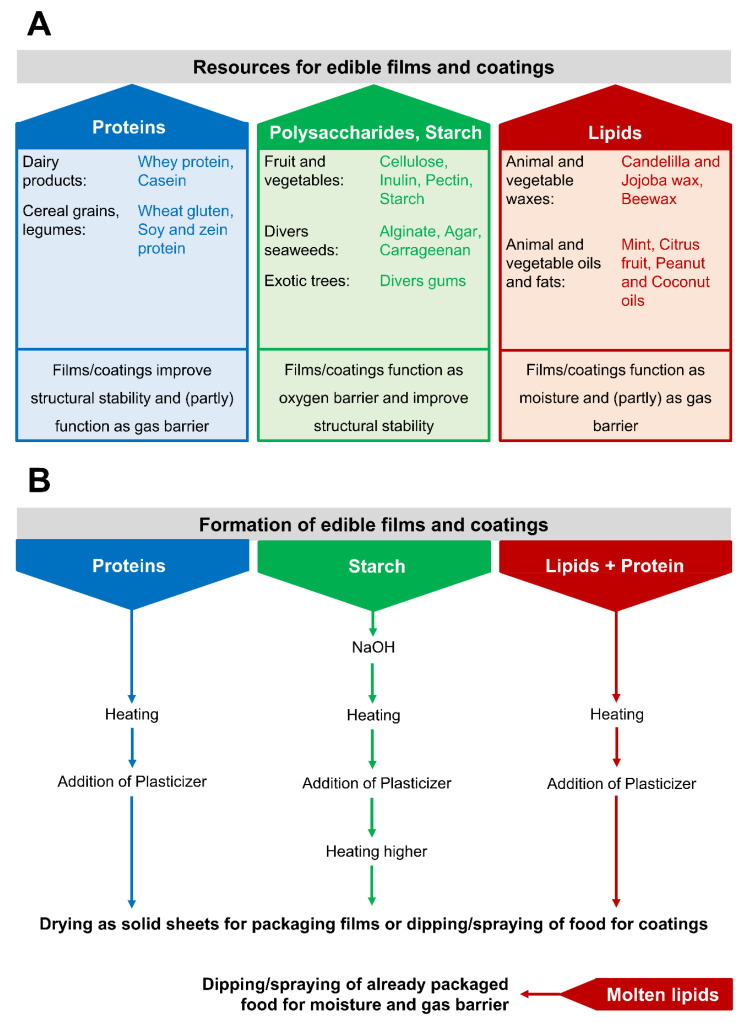
Use of food wastes and by-products for food packaging. (**A**) Food waste resources for edible films and coatings and their preferred application in food packaging (modified and extended according to Janjarusskul et al. [81], Rawdkuen et al. [87], and Huber et al. [88]). (**B**) Formation of edible films and coatings based on the recycling of starch, proteins, and/or lipids.

The majority of the above-mentioned coating materials can be obtained from food wastes or by-products, which still consist of organic compounds and are divided into proteins, polysaccharides, starch, and lipids [81,87]. In Figure 1A the most commonly used materials and their origins are shown, whereby each group of compounds possesses specific chemical properties, resulting in different applications for food packaging. Protein-based coatings are mainly used to achieve higher stability of foods or to reduce oxidation processes and loss of aroma over time, although the suitability as a gas barrier depends on the outer environment [81,83,84,88]. Thus, the oxygen permeability of whey-protein-coated plastic films was significantly reduced compared to non-coated films but showed an exponential temperature and humidity-dependent increase in gas exchange [84]. The gas permeability of protein-based packaging is also influenced by the addition of plasticizers during film formation [89] (Figure 1B). At first, the proteins were heated to unfold them and induce the formation of irreversible aggregates by the generation of new disulfide bonds and further intermolecular interactions [90,91]. Without this heating step, the final film becomes brittle and cracks easily while drying because of the missing interactions between the molecules [92]. Moreover, due to the disulfide bonds, the protein-based films become insoluble in water [93]. After heating, plasticizers are added to the protein solution, increasing the intermolecular space between the proteins and resulting in higher flexibility of the film as well as a higher gas permeability [91]. Finally, the liquid coating solution is dried in the dehydration step, while a complex network of proteins and plasticizers is formed [91]. The application as a food packaging takes place during the dehydration step by dipping or spraying; then it is termed as a coating, or alternatively the prepared solution is dried as solid sheets prior to packaging, which is termed as a film [92,93]. Similar to the formation of protein-based films and coatings, starch needs to be heated, which induces irreversible structural transitions [94]. Firstly, starch is mixed with water and NaOH, whereby the water leads to swelling of the starch granules, while NaOH improves the final flexibility of the packaging [95,96]. Then, the solution is heated, which induces the gelatinization of the starch [97,98,99]. In this step, the starch absorbs water, swells, and loses its crystallinity and birefringence, leading to structural transitions [94,95,96,97,98]. After that, the temperature is further increased, followed by the addition of a plasticizer, which increases the flexibility and water permeability of the coating, until the solution is finally dried for film formation [95,96]. Starch- and cellulose-based films can protect the food against mechanical damage, while the tensile stress and hardness of the film correlates with the included cellulose concentration [99]. In contrast, lipid-based films and coatings are mostly used as an effective moisture barrier, reasoned by their low water affinity and the restricted moisture permeability [100]. Nevertheless, such lipid-based coatings are often combined with protein solutions due to the high inflexibility of lipids used alone as coatings or films [100]. Depending on the prepared protein film/solution and the followed application, three different processes for formation have already been established: (I) the protein solution is mixed with plasticizer and a lipid fraction, followed by heating and homogenization [101,102,103,104]; (II) one or more lipid layers are additionally added by immersion of the food in molten lipid [105]; (III) molten lipid is brushed onto protein films, e.g., as a further moisture barrier [106]. 

### 4.4. Active Packaging

While the use of edible films and coatings based on food wastes has been more focused, the development of a combination of edible and active packaging has also been promoted. In this kind of coatings, bioactive compounds obtained from material considered as waste are incorporated, thus giving added value to these products [21]. Such beneficial values could be antimicrobial and/or antioxidant effects, as well as the generation of CO_2_ by specific emitters to maintain a stable atmosphere for longer shelf-life [107]. An increased consumer demand regarding food safety also clarifies the high priority on developing active packaging for slowing down the processes of food spoilage, which may cause food poisoning [108]. The shelf-life of food is mainly affected by bacterial growth or lipid oxidation, which promotes the incorporation of antibacterial compounds and antioxidants into packaging films and coatings [108]. Han et al. [109] developed a peanut coating based on whey protein isolate including up to 1% of α-tocopherol and ascorbyl palmitate as antioxidants. While the whey protein coating significantly reduced the oxidation and served as an effective oxygen barrier, the antioxidants showed no further effect on food oxidation. Nevertheless, by adding ascorbyl palmitate into the coating solution, its viscosity was remarkably improved [109]. Similar results were shown by Min and Krochta [110] by incorporating ascorbic acid into whey protein coating as an oxygen scavenger. Ascorbic acid significantly reduced the oxygen permeability and the following lipid oxidation compared to non-coated peanuts, but these effects were mainly induced by the coating rather than the antioxidant. Hence, no difference in lipid oxidation was documented between the coatings with and without ascorbic acid [110]. As already mentioned, the second most interesting point in active packaging is the development of antimicrobial coatings. Food poisoning and other foodborne illness are mainly caused by *Listeria monocytogenes*, which is able to survive and grow in the refrigerator at 2–4 °C [107]. Therefore, antimicrobial packaging may prevent listeriosis and could improve food safety, especially in the case of ready-to-eat products [107]. Corrales et al. [51] analyzed the antimicrobial effects of a grape seed extract against several bacteria, e.g., *Listeria monocytogenes*, followed by combining the extract with an aqueous pea starch coating solution. Although the extract showed a dose-dependent increase in bacterial growth inhibition, only 8% of the total phenolics, occurring in the food coating, migrated to the surface of the packed meat. However, the growth of the bacteria strain *B. thermosphacta* could be significantly reduced by the active packaging after 4 days of storage [51]. A combination of antioxidant- and antimicrobial-affecting packaging was developed by Kang et al. [52], who enriched a pectin-based coating solution with green tea extract. While the pectin coating reduced the bacteria content in the samples, the antimicrobial potential of the incorporated green tea extract tendentially increased this effect and simultaneously led to a significantly lower lipid oxidation [52]. 

### 4.5. Intelligent Packaging

**Figure 2:**
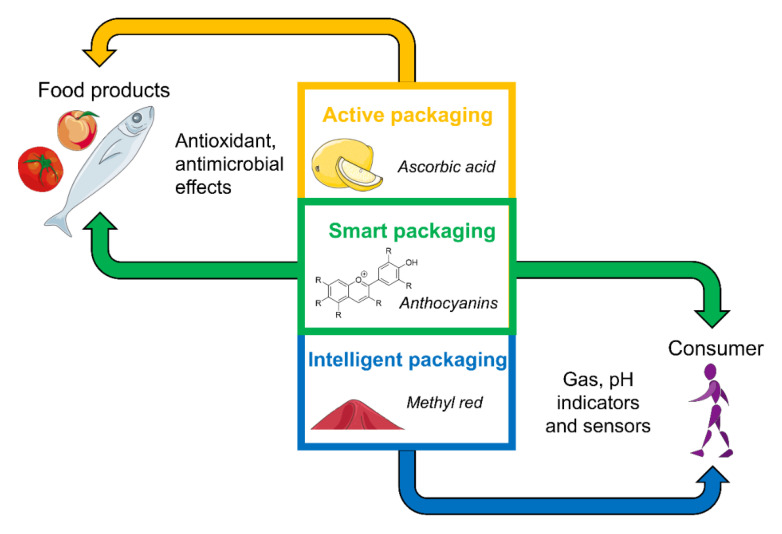
Differentiation of active, intelligent, and smart packaging according to their influences on food products and consumers. This figure was created using templates from Servier Medical Art (https://smart.servier.com (accessed on 28 June 2021)), which are licensed under a Creative Commons Attribution 3.0 Unported License.

Another use of food waste and by-products is their application as dyes for intelligent packaging, which has recorded a constant increase in interest and publications in recent years [111]. Compared to the usually-used foils and coatings, intelligent packaging enables the observation of the food and the environmental conditions like temperature or the packaging atmosphere, simultaneously giving information on the shelf-life and edibility of the product to the consumer [87,111,112]. Moreover, this new technique is more reliable than the expiry date, reasoned by early detecting damaged packaging and therefore warning the consumer in case of a lower shelf-life of the product [112]. These advantages as well as the principle of intelligent packaging must not be confused with the technique of active packaging, although both can be summarized with the term smart packaging [111] (Figure 2). While active packaging has a specific function on the foodstuff and protects it against influencing factors, e.g., oxidative processes, intelligent packaging measures such changes and gives this information to the consumer without influencing the food [111,112]. This kind of ‘communication’ with the consumer occurs by a visual modification, mainly shown as color changes of sensitive dyes, whereby different colors represent freshness or food spoilage [112]. The focus of intelligent packaging is on the detection of gas molecules (H_2_, O_2_, NO_2_, and CO_2_) and the pH level, all factors which cannot be seen directly by the consumer and especially not if the packaging is still closed [87,112]. The above-mentioned factors correlate with microbial growth, metabolites of bacteria, or the temperature and therefore enable the observation of the food shelf-life from packaging, from transportation up to opening [53,113]. However, depending on the freshness factor, the measuring receptors are labelled on the outside of the packaging, e.g., for measuring the temperature or alternatively for detecting pH and atmosphere changes, they are attached inside the packaging solution by having contact with the atmosphere or food [87]. For intelligent packaging, different systems have been developed: (I) sensors, which only measure the quality factors and need a separate device for signal transducing; and (II) indicators, which measure the spoilage progress and visualize all information directly, independent of any external support [87,114]. The latter works with chemical, color-changing dyes, which may be substituted by natural extracts originated in food wastes and/or by-products [87]. Bromothymol blue (shifting from blue to yellow by a decrease in pH) and methyl red (shifting from yellow to red) are used as chemical dyes in intelligent packaging [115]. By incorporating these dyes in a cellulose-based coating solution, which is attached to the inside of packaging, a fast and sensitive method for detecting CO_2_ as spoilage metabolite was established in several studies [115,116]. Nevertheless, chemical dyes are made synthetically, and a possibly toxic potential on consumers’ health prohibits their use in food applications, whereas natural compounds may represent a safe and suitable alternative [87]. One of these natural compounds with color-changing properties are anthocyanins, which occur in several types of fruits as well as in their wastes and by-products [117]. Especially, winery waste material like grape skin and peel, or, e.g., waste of purple corncobs, represents anthocyanin-rich resources for the generation of new food coatings [117,118,119]. Luchese et al. [120] aimed to reuse anthocyanin-rich industrial waste for intelligent packaging and analyzed the suitability of blueberry powder as a pH-sensitive indicator. The powder was incorporated in corn starch-based films, followed by incubation of the film in buffer solutions, finally showing a broad range of pH-induced color changes [120]. At acidic pH, the film appeared rose, changed to red and purple at neutral pH, and becoming grey, black, and brown with increasing pH value. All these modifications in color are perceptible to humans, enabling a reliable detection of food shelf-life [120]. An equally efficient indicator was generated by adding anthocyanins to a chitosan packaging solution, whereby the color range was slightly different and changed from pink as the acidic form to green and yellow as the basic form [121]. Pereira et al. [53] went one step further by extracting anthocyanins from red cabbage, incorporating the extract in a PVA/chitosan hydrogel and finally testing the film as an indicator for milk spoilage. Fresh milk has an average pH of 6.7, shown by a grey indicator color, while microbial growth metabolites reduce the pH to 4.6, which led to a pink color of the film [53]. To sum up, anthocyanins are a reliable pH-sensitive indicator for monitoring food quality and shelf-life [113] and could be easily extracted from food wastes, contributing to the actual trend of recycling. Furthermore, anthocyanins are widely known for their high antioxidant potential [122]. According to this, Gil et al. [123] showed that anthocyanins are one of the most effective antioxidants in pomegranate juice. Moreover, anthocyanin-enriched extracts and juices successfully protect cells or human against oxidative stress in several experiments [124,125,126,127]. Due to the pH-sensitive color changes and the antioxidant effects of anthocyanins, these compounds seem to be the optimal ingredient to combine intelligent and active packaging rolled into one. Hence, in several studies the combined advantages of using anthocyanins as radical scavenger and spoilage indicator in food packaging was analyzed [54,128,129]. Yun et al. [128] generated starch films including different concentrations (0–4 wt %) of anthocyanins, whereby the concentration significantly influenced the documented results in a dose-dependent manner. While all films showed a pH-induced color variation of the film, the lowest concentration of 1 wt % enabled a better visual differentiation of the color changes [128]. On the contrary, the higher the anthocyanin concentration in the film was, the higher was the scavenging activity with up to 75% against DPPH radicals [128]. Similar results with a radical scavenging activity of up to 81% were reported by Qin et al. [54], analyzing the antioxidant and pH-sensitive potential of a cassava starch-based film containing anthocyanins. The positive correlation of the anthocyanin concentration with the antioxidant potential, as well as the better suitability of a lower-concentrated film as freshness indicator was identical for both Yun et al. [128] and Qin et al. [54]. The highest antioxidant potential was measured by Uranga et al. [129]. While more than 95% of the anthocyanins, extracted from red cabbage and mixed with fish gelatine during the film formation process, were released within two days of immersion in an ethanol solution, more than 91% of DPPH radicals was inhibited by the packaging film [129]. Compared to α-tocopherol, a widely used natural antioxidant, anthocyanins showed a similar radical scavenging activity [129] and represent an effective ingredient in active packaging. Conclusively, anthocyanins maintain their pH-sensitive and antioxidant properties during film processing and are therefore excellent compounds for combining both active and intelligent food packaging [128,129]. Furthermore, anthocyanins are naturally occurring compounds that could be utilized in edible films and coatings likewise.

## 5. Conclusions

The utilization of waste and by-products from the agro-industry and food processing have gained attention in recent years. In this review, potential applications of fruit- and vegetable-based by-products as biopolymers, biocomposites, active or intelligent packaging as well as edible films and coatings have been highlighted together with the advantages, disadvantages and applications. Bio-based safe, value-added, green materials exhibit promising properties such as biodegradability, renewability, reduced production cost, decreased waste and environmental impact. Therefore, future work should focus on the treatments or additives to improve the thermal resistance, barrier and mechanical properties of related biomaterials.

## Figures and Tables

**Table 1:** Application of fruit and vegetable wastes and by-products in food packaging systems.

Waste/By-Product	Packaging System	Applied Food Product	Packaging Properties	Reference
Pomegranate peel (PPE)	Active packaging—PPE at different concentrations (0, 25, 50, 75 mg/mL of film forming solution) added to zein films	Kalari cheese	inhibition of all target pathogens, ↑ antioxidant and antimicrobial activity, delay of oxidation, improved film flexibility, ↑ tensile strength	[41]
Pomegranate peel extract (PPE)	Chitosan (1% *w*/*v*) and alginate (2% *w*/*v*) edible coatings + PPE (1% *w*/*v*)	Guava	improved postharvest guava quality, delayed senescence, ↓ respiration rate, retarded oxidation, ↑ phenolic content and ascorbic acid levels	[42]
Tomato by-product extract (TBE)	TBE added to films containing poly(vinyl alcohol) (3% wt/v) and chitosan (1% wt/v)	-	improved antibacterial activity toward *S. aureus* and *P. aeruginosa*, ↑ resistance of films, ↑ antioxidant activity	[43]
Red grape seeds, white grape seeds, tomato waste extracts	stabilizers added to polypropylene (PP) films	-	red grape seed: ↑ PP stabilization and ↓ oxidation, greater than tomato extracts	[44]
Mango peel and seed kernel	Edible film-containing mango peel flour (1.09%) and glycerol (0.33%), and extract of mango seed (0.078 g/L)	Peach	↑ permeability, antioxidant activity, hydrophobicity, and surface properties, 39% less O_2_ consumption, 64% less ethylene production and 29% less CO_2_ production	[45]
Potato peel	Bioactive film—potato peel at different ratios of potato cull (0, 0.5, 1, and 1.3 g peel/g cull)	-	improved elongation and dose dependently, ↑ antioxidant activity, ↑ tensile strength, ↓ film solubility, ↓ moisture and water activity	[46]
Banana peel extract (BPE)	Chitosan composite film—BPE at concentrations 4%, 8%, and 12%	Apple	best results with 4% BPE, ↑ thickness, ↓ moisture content, ↓ water vapor permeability, improved mechanical properties, and postharvest apple quality	[47]
Blueberry leaf extract (BLE)	Chitosan coating + BLE (4%, 8%, 12%)	Blueberry	↓ decay of the fruit, ↓ weight loss, inhibition of target pathogens, ↑ total phenolic content	[48]
Grapefruit seed extract (GSE)	Carrageenan-based antimicrobial film + GSE at 0.6, 3.3, 6.6, 10, and 13.3 g/mL	-	↑ water vapor permeability, ↓ tensile strength, ↑ elongation, inhibition of tested pathogens, improved UV barrier property	[49]
Apple peel polyphenols (APP)	Chitosan film + APP at 0.25, 0.50, 0.75, and 1.0%	-	↑ thickness, density, swelling degree, solubility and opacity, improved water barrier property, ↑ antioxidant property, inhibition of tested pathogens	[50]
Grape seed extract (GSE)	Pea starch (3% *w*/*w*) and glycerol (1.8%) + 1% GSE	-	↑ thickness, oxygen permeability, ↓ tensile strength, ↑ antibacterial effect by phenolic acids	[51]
Green tea extract (GTE)Pectin from citrus fruits	Pectin and polyethylene glycol + 0.5 g/100 mL GTE	Pork patties	↓ lipid peroxidation, ↑ radical scavenging activity, ↑ antibacterial effect	[52]
Red cabbage	PVA/chitosan hydrogel + 25% (*v*/*v*) anthocyanin extract from red cabbage	Milk	pH-sensitive color change for spoilage detection	[53]
*Lycium ruthenicum* Murr (LR)	Starch and glycerol + 0, 1, 2, and 4 wt % anthocyanins from LR	Pork	↑ thickness, ↓ moisture content, ↑ scavenging activity, pH-sensitive color change for spoilage detection	[54]
Red cabbage	PVA/starch solution + 23% (*v*/*v*) anthocyanin extract from red cabbage and boric acid and 0.5, 2, 5, 10, and 20% propolis extract	-	↑ tensile strength, ↑ moisture retention, ↑ antibacterial effect, pH-sensitive color change for spoilage detection	[55]
